# An investigation of the impact of using different methods for network meta-analysis: a protocol for an empirical evaluation

**DOI:** 10.1186/s13643-017-0511-x

**Published:** 2017-06-24

**Authors:** Amalia (Emily) Karahalios, Georgia Salanti, Simon L. Turner, G. Peter Herbison, Ian R. White, Areti Angeliki Veroniki, Adriani Nikolakopoulou, Joanne E. Mckenzie

**Affiliations:** 1 School of Public Health and Preventive Medicine, Monash University, level 1, 549 St Kilda Road, Melbourne, Victoria Australia; 2 Institute of Social and Preventive Medicine (ISPM), University of Bern, Bern, Switzerland; 3 University of Otago, Dunedin, New Zealand; 4 MRC Biostatistics Unit, Cambridge, UK; 5 MRC Clinical Trials Unit at UCL, London, UK; 6 Li Ka Shing Knowledge Institute, St Michael’s Hospital, Toronto, Ontario Canada

**Keywords:** Network meta-analysis, Mixed-treatment comparison, Indirect treatment comparison, Heterogeneity, Arm-based, Contrast-based, Bayesian, Empirical evaluation, Evidence-based methods, Multiple treatment comparison

## Abstract

**Background:**

Network meta-analysis, a method to synthesise evidence from multiple treatments, has increased in popularity in the past decade. Two broad approaches are available to synthesise data across networks, namely, arm- and contrast-synthesis models, with a range of models that can be fitted within each. There has been recent debate about the validity of the arm-synthesis models, but to date, there has been limited empirical evaluation comparing results using the methods applied to a large number of networks. We aim to address this gap through the re-analysis of a large cohort of published networks of interventions using a range of network meta-analysis methods.

**Methods:**

We will include a subset of networks from a database of network meta-analyses of randomised trials that have been identified and curated from the published literature. The subset of networks will include those where the primary outcome is binary, the number of events and participants are reported for each direct comparison, and there is no evidence of inconsistency in the network. We will re-analyse the networks using three contrast-synthesis methods and two arm-synthesis methods. We will compare the estimated treatment effects, their standard errors, treatment hierarchy based on the surface under the cumulative ranking (SUCRA) curve, the SUCRA value, and the between-trial heterogeneity variance across the network meta-analysis methods. We will investigate whether differences in the results are affected by network characteristics and baseline risk.

**Discussion:**

The results of this study will inform whether, in practice, the choice of network meta-analysis method matters, and if it does, in what situations differences in the results between methods might arise. The results from this research might also inform future simulation studies.

## Background

Network meta-analysis (NMA) (also referred to as mixed treatment comparisons or multiple treatment comparisons) is the quantitative component of a review that combines direct and indirect evidence across a network of treatments [1]. NMA has many potential benefits compared with pairwise meta-analysis, with one particular advantage being the ability to rank a set of competing treatments according to their safety or effectiveness, or both, thus facilitating clinical decision-making [2]. The use of NMA methods has become increasingly common [3], alongside methodological developments (see, for example, Efthimiou et al. [4] for a recent review of NMA methodology), tutorial papers explaining the methods (e.g. [5, 6]), and user written packages/routines in Stata, R, and WinBUGS/OpenBUGS (e.g. [4, 7]).

Two broad approaches have been proposed for the synthesis of evidence across networks. In the first approach, the trial-specific relative treatment effects (e.g. log of the odds ratio) over the trials are pooled (henceforth referred to as contrast-synthesis models), whereas in the second approach, the absolute estimates (e.g. log odds) of each arm are pooled and treatment effects (e.g. odds ratios, risk differences) are constructed from the arm estimates (henceforth referred to as arm-synthesis models) [8]. Detailed explanations of these models have been published [2, 8–11] and brief explanations are provided below. There has been recent debate about the validity of the arm-synthesis models [8, 10, 12]; proponents argue that they offer an advantage to standard methods because they allow the analyst to estimate both relative and absolute measures [13]. However, opponents argue that these methods represent a departure from standard meta-analysis practice as they compromise the advantage of randomisation by relating arms across studies [8].

A common assumption that is made under the random-effects contrast-synthesis model is that the between-trial heterogeneity variance of the relative treatment effects is the same for every treatment comparison [2, 4]. This assumption, referred to as the ‘homogeneous variance assumption’ [14] reduces the number of parameters that need to be estimated, simplifies the estimation and increases the precision for estimating the heterogeneity variance [4]. However, even with this assumption, the available data in the network may be limited for estimating the heterogeneity variance. The Bayesian framework for fitting the contrast-synthesis model offers the opportunity of incorporating external estimates of heterogeneity, such as those estimated from large empirical data sets, which may improve precision in the estimation of the heterogeneity variance [15]. Turner et al. [16] provide informative prior distributions defined by outcome type and intervention comparison [16, 17].

Evidence about the relative merits and drawbacks of statistical methods comes from statistical theory, numerical simulation studies, and empirical evaluation. Simulation studies allow investigation of the performance of statistical methods against a known truth [18], and are useful for exploring the properties of the methods (e.g. Song et al. [19]), particularly in scenarios where the assumptions of the underlying methods may not be met. Empirical studies allow investigation of how methods work in practice using actual data (e.g. Langan et al. [20]), and in particular, allow estimation of the magnitude of discordance in results between the methods, and exploration of factors that might predict this discordance. To facilitate both simulation studies and empirical research on NMA methods, a dataset of 456 NMAs containing available data from all published NMAs since 1999 has been compiled [21].

To our knowledge, no study has empirically compared the results of NMA between the contrast-synthesis and arm-synthesis models across a large number of networks. In this study, we will achieve this through the re-analysis of published networks of interventions with binary outcomes using five NMA models.

## Methods/design

### Database of networks

We will use a database of 456 published NMAs of randomised trials that have been previously identified and curated. Details of the methods for locating the NMA publications, inclusion criteria, and the screening process are outlined in Petropoulou et al. [21]. The database compares networks with at least four different interventions that employed an appropriate synthesis method. The data extracted from the networks (of importance for the present study) included publication characteristics (e.g. year of publication, journal of publication); summary statistics (e.g. number of events and number of participants per arm) from the direct comparisons of the primary NMA outcome in the review (or the outcome identified from a decision tree when no primary outcome was specified [21]; classification of the outcome in terms of whether it was harmful or beneficial, and whether it was an objective, semi-objective, or subjective measure; and whether the type of included treatment comparisons were pharmacological vs placebo, pharmacological vs pharmacological or non-pharmacological vs any intervention.

### Eligibility criteria for the present study

We will include a subset of networks from the database which meet the following inclusion criteria: (i) a binary primary outcome, (ii) the number of events and number of participants are available for each trial arm, and (iii) for networks including at least one closed loop, there is no evidence of statistical inconsistency as detected by a *p* value <0.10 via the design by treatment interaction test using Stata’s **mvmeta** command [22]. Tests for the evaluation of the consistency of a network, for example, the design by treatment interaction, are known to have low power, which led us to choose a *p* value of 0.10 as our cut-off [19, 23].

### Statistical methods to analyse the networks

We will begin by describing the notation, then describe the contrast-synthesis models, followed by a brief description of the arm-synthesis models. Details about the estimation methods and packages used to fit the models in R are described in the section ‘Implementing the models in R’ below.

In the following, we assume that there are *I* studies in the network (labelled *i* = 1, 2, …, *I*) and that each study investigates a subset of the *K* treatments. The studies are labelled *k* = 1, 2, …, *K* and the subset of treatments compared in study *i* is denoted *S*_*i*_. We further assume that in study *i*, the observed number of events, *y*_*ik*_, has arisen from a binomial process, *y*_*ik*_ *~* bin(*n*_*ik*_ *,p*_*ik*_), where *n*_*ik*_ is the number of participants in arm *k* $\left( k\in {S}_i\right)$, and *p*_*ik*_ is the probability of the event.

#### Contrast-synthesis models

In the contrast-synthesis model, a baseline treatment *b* (usually placebo or standard of care) is specified for each study $i$. The hierarchical model for this approach is

$$ g\left({p}_{i k}\right) = {\mu}_i+{X}_{i k}{\delta}_{i bk};\kern1.25em  k\in {S}_i $$

$$ {\delta}_{ibk}\sim N\left({\theta}_{bk},{\tau}_{bk}^2\right); $$

where $g\left(\bullet \right)$ is the logit link, *μ*_*i*_ are the study-specific baseline effects and will be treated as unrelated nuisance parameters, *δ*_*ibk*_ represents the study-specific effect of treatment *k* relative to the baseline *b* and *X*_*ik*_ is an indicator variable which is set to 0 if *k* = *b* and 1 otherwise. The study-specific treatment effects *δ*_*ibk*_ are drawn from a common random-effects distribution, where *θ*_*bk*_ represents the mean effect of treatment *k*, relative to the baseline treatment, and *τ* ^*2*^_*bk*_ represents the between-trial variance in treatment effects. Finally, we make the consistency assumption that if any two treatments, say *x* and *y*, are compared indirectly through *b*, the result will be consistent with the direct comparison; that is: *θ*_*xy*_ *= θ*_*bx*_ *− θ*_*by*_. For multi-arm trials, an adjustment is required where *δ*_*ibk*_ is assumed to be multivariate normally distributed [2]. Implementation of models with a binomial likelihood is possible in either a frequentist or Bayesian framework but these are easier to estimate in a Bayesian framework [2]. For this reason, we will use a Bayesian framework to fit the variants of this model (see the section ‘Contrast-synthesis models in a Bayesian framework’).

An alternative contrast-synthesis approach is to model the estimates of the treatment contrasts. First, let ${Y}_{ib k}= log\left(\frac{p_{ik}/\left(1-{p}_{ik}\right)}{p_{ib}/\left(1-{p}_{ib}\right)}\right)$ denote the log odds ratio for treatment *k* relative to the baseline *b* in trial *i*, the model here is then:

$$ {Y}_{ibk}\sim N\left({\delta}_{ibk},{\sigma}_{ibk}^2\right) $$

where ${\sigma}_{ibk}^2$ is the variance of the log odds ratio and ${\delta}_{ibk}$ is drawn from the common random-effects distribution above. We will implement this model in a frequentist framework (see section ‘Contrast-synthesis model in a frequentist framework’).

#### Arm-synthesis models

The arm-synthesis model is specified such that:

$$ {\varPhi}^{-1}\left({p}_{i k}\right) = {\mu}_k+{v}_{i k};\kern1.25em  k\in {S}_i $$

$$ {\left({v}_{i1},{v}_{i2},,\dots, {v}_{i K}\right)}^T\sim M V N\left(\mathbf{0},{\boldsymbol{\Sigma}}_{\boldsymbol{K}}\right); $$

where $\varPhi \left(\bullet \right)$ is the standard normal cumulative distribution function. The log link function can also be used [10]. The *μ*_*k*_’ s are fixed effects for the treatments, and the random effects ${v}_{iK}$ are correlated within each study with the covariance matrix ${\boldsymbol{\varSigma}}_{\boldsymbol{K}}$. Various assumptions can be made about the variance-covariance matrix, which allow for different correlations across the studies. Consistency on the probit scale is implicit in this model. For further details of this model, the reader is referred to Zhang et al. [13, 24]. We will implement this model in a Bayesian framework (see the section ‘Arm-synthesis models in a Bayesian framework’).

### Implementing the models in R

#### Contrast-synthesis models in a Bayesian framework

We will fit two contrast-synthesis models in a Bayesian framework using the R package **gemtc** https://cran.r-project.org/package=gemtc (Table 1). For both models, we will fit a random-effects consistency model, with a binomial likelihood and logit link function, and a variance-scaling factor of 2.5. We will use two different prior distributions for the between-trial heterogeneity standard deviation (*τ*): a vague prior uniform distribution, where $\tau \sim \mathrm{Uniform}\left(0,10\right)$ [25] (referred to as contrast-synthesis model 1) and an informative prior distribution (referred to as contrast-synthesis model 2). The informative prior distribution will be selected from the predictive distributions for between-trial variance available in Turner et al [16]. These distributions are specific to outcome types (all-cause mortality, subjective, semi-objective) and treatment comparisons (pharmacological, non-pharmacological, placebo/control). Our networks have been classified using the same categorisation as in Turner et al. [16]. Specifically, in the presence of placebo in the network, the network was categorised as pharmacological vs placebo. If only pharmacological treatments were available, then the network was categorised as pharmacological vs pharmacological, whereas if a non-pharmacological treatment was included in the network, then the network was categorised as non-pharmacological vs any category. For the contrast-synthesis models 1 and 2, we will assume a common estimate for the between-trial heterogeneity variance across the different treatment comparisons [11].

**Table 1:** Overview of the methods applied to synthesise the evidence from network meta-analyses

Method label	Package used in R	Contrast-level or arm-level input data	Frequentist or Bayesian framework	Likelihood and link functions	Heterogeneity	Prior distributions		
						Treatment-specific fixed effects	Mean effect of treatment k relative to baseline	Heterogeneity or random effects parameter
Contrast-synthesis model 1	gemtc (version 0.8.1)	Arm-level	Bayesian	Binomial likelihood and logit link	Homogeneous/common	N/A	*δ*_*k*_ ~ *N*(0, (15*5)^2^)^a^	*τ*_*bk*_ ~ *U*(0,10)^b^
Contrast-synthesis model 2	gemtc (version 0.8.1)	Arm-level	Bayesian	Binomial likelihood and logit link	Homogeneous/common	N/A	*δ*_*k*_ ~ *N*(0, (15*5)^2^)^a^	Informative^c^
Contrast-synthesis model 3	netmeta (version 0.9-2)	Contrast-level	Frequentist	N/A	Homogeneous/common	N/A	N/A	N/A
Arm-synthesis model 1^d^	pcnetmeta (version 2.4)	Arm-level	Bayesian	Binomial likelihood and probit link	Homogeneous/common	*μ*_*k*_ ~ *N*(0, 1000)	N/A	*σ*_*k*_ ~ U(0,10)
Arm-synthesis model 2^e^	pcnetmeta (version 2.4)	Arm-level	Bayesian	Binomial likelihood and probit link	Heterogeneous	*μ*_k_ ~ *N*(0, 1000)	N/A	*σ*_k_ ~ U(0,10)

*N/A* not applicable

^a^Source: documentation for gemtc package https://cran.r-project.org/package=gemtc

^b^ *τ*_bk_ represents the between-trial heterogeneity standard deviation in treatment effects

^c^Each network was categorised according to the type of its included treatment comparisons and outcomes [21]. Specifically, in the presence of placebo in the network, the network was categorised as pharmacological vs placebo. If only pharmacological treatments were available, then the network was categorised as pharmacological vs pharmacological, whereas if a non-pharmacological treatment was included in the network, then the network was categorised as non-pharmacological vs any category. Outcomes were categorised as all-cause mortality, subjective, or semi-objective. The predictive distributions for between-trial heterogeneity variance for each of the treatment comparison by outcome type categories, estimated in Turner et al. [16], were used as informative priors

^d^Model assumes homogeneity of the variances (i.e. common variance) of the random effects and assumes that the off-diagonal elements of the correlation matrix are equal (specified by the hom_eqcor option)

^e^Model assumes an unstructured covariance matrix of the random effects and assumes that the off-diagonal elements of the correlation matrix are equal (specified by the het_eqcor option)

#### Contrast-synthesis model in a frequentist framework

We will fit a contrast-synthesis model in a frequentist framework using the R package **netmeta** https://cran.r-project.org/package=netmeta (Table 1). The approach implemented in this package is based on weighted least squares estimation. The approach was first developed using graph-theoretical methods that derive from electrical network theory. For a detailed description of the method and its derivation from graph-theoretical methods, the reader is referred to Rücker [26] and Chapter 8 of Schwarzer et al.’s textbook *Meta-analysis with R* [27]. We refer to this model as contrast-synthesis model 3. The data will be input as contrast-level data, and the **pairwise** function in the **netmeta** package will be used to convert arm-level data to contrast-level data.

#### Arm-synthesis models in a Bayesian framework

We will fit two arm-synthesis models in a Bayesian framework using the R package **pcnetmeta** https://CRAN.R-project.org/package=pcnetmeta (Table 1). For both models, the correlations between the random-effects within studies are assumed equal. We will fit a model with homogeneity of variances of the random-effects (referred to as arm-synthesis model 1), which is implemented by setting the model argument of **pcnetmeta** equal to **hom_eqcor**. Next, we will fit a model with an unstructured heterogeneous variance of the random-effects (referred to as arm-synthesis model 2), which is implemented by setting the model argument of **pcnetmeta** equal to **het_eqcor**. We will use a uniform prior distribution for the standard deviations ($\sigma$) of the random-effects ${v}_{ik}$ above, $\sigma \sim U n i f o r m\left(0,10\right)$.

For the Bayesian models (i.e. contrast-synthesis models 1 and 2 and arm-synthesis models 1 and 2), three chains will be used with a burn-in of 300,000 followed by 300,000 samples saved at an interval of 10 from each of the three chains [28]. Convergence will be assessed by the Brooks–Gelman–Rubin method [29, 30] and by visual inspection of the history plots [31].

### Network estimates

Analysing each NMA using the five methods described above (contrast-synthesis models 1–3 and arm-synthesis models 1–2), we will estimate the log of the odds ratios and their corresponding standard errors for each pairwise comparison within a network; the rank of each treatment based on the surface under the cumulative ranking (SUCRA) curve [31]; the corresponding SUCRA value (the *p*-score for the contrast-synthesis model in a frequentist framework); and the between-trial heterogeneity variance for the contrast-synthesis models.

### Differences in the network estimates between the methods

We will use the following metrics to compare the network estimates (described in the ‘Network estimates’ section) between the five methods:

1. Raw difference in the log of the odds ratio
2. Ratio of standard errors of the log of the odds ratios
3. Difference in rank based on the SUCRA value
4. Difference in SUCRA value
5. Ratio of the estimates of the between-trial heterogeneity variance for the contrast-synthesis methods.

### Exploring factors that might affect the differences in network estimates between the methods

The differences in the estimates between the NMA methods might be modified by several factors including the size of the network, the rareness of events, and the distribution of information in the network. The size of a network and the distribution of information within a network might be determined by the number of studies, the number of treatments, or the number of nodes/comparisons, and their ratios. Therefore, we will investigate whether the following three metrics modify the differences in the network estimates: the ratio of the number of treatments to the number of studies, the ratio of the number of treatments to the number of available direct comparisons, and the ratio of the number of studies to the number of available direct comparisons. We will also investigate if the proportion of arms in a network with less than 10 events modifies differences in the network estimates.

### Statistical analysis

**Fig. 1:**
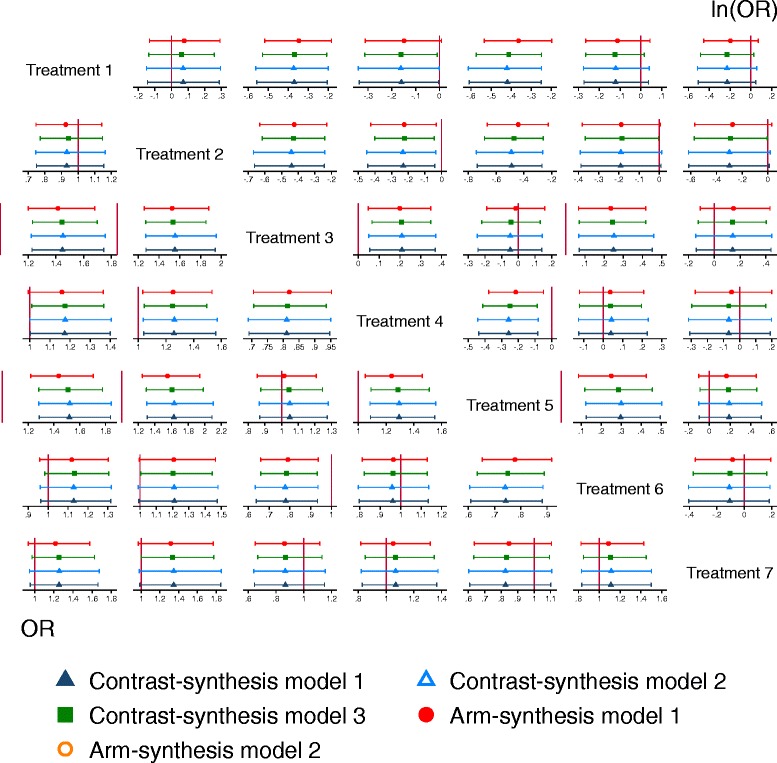
Log of the odds ratio and corresponding 95% confidence/credible interval for each pairwise comparison within one network. Note that the data pictured is from a network that is not included or related to the networks in our

We will begin by visually inspecting the effect estimates and confidence/credible intervals for each pairwise comparison within each network (Fig. 1).

We will fit multilevel models, including random effects for network and comparison to estimate differences in the metrics (e.g. log(ORs), ranks) between the methods. The initial model will include only a term for method, and additional models will include factors hypothesised to modify differences in the network estimates between the methods. We will use the contrast-synthesis model 1 as the reference method.

### Graphical methods

**Fig. 2:**
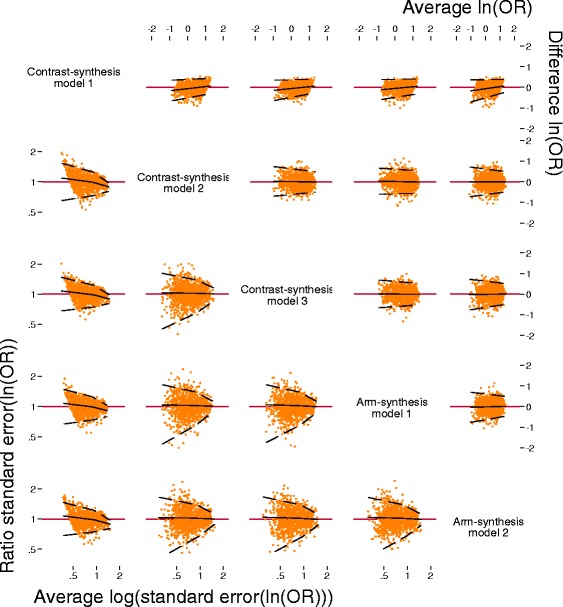
Bland-Altman plots for the level of agreement between the log of the odds ratios (*top right*) and standard errors for the log of the odds ratios (*bottom left*) comparing the five methods used to synthesise evidence from network meta-analyses. Note that the data pictured have been simulated for illustrative purposes

**Fig. 3:**
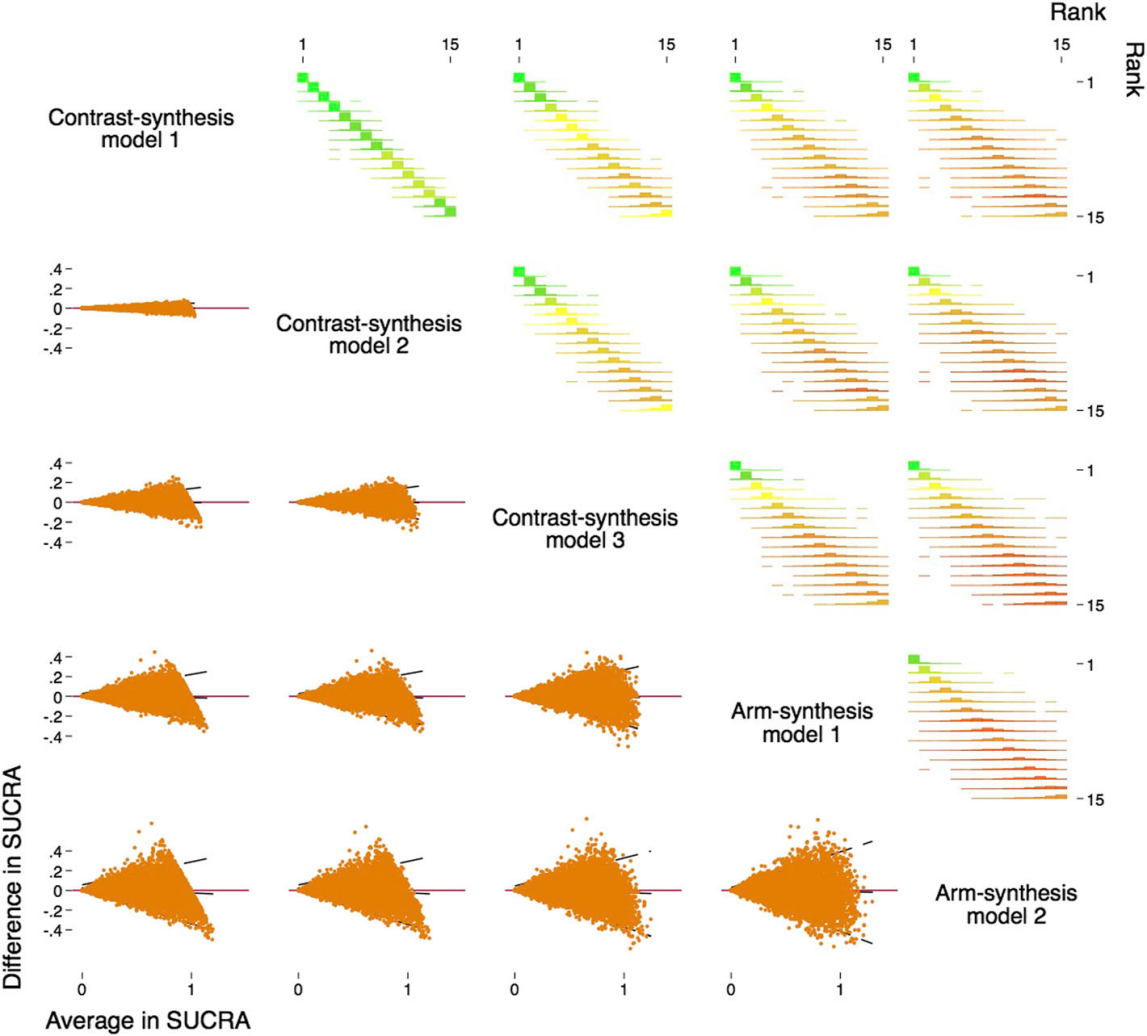
Comparison of ranks (*top right*) and Bland-Altman plots for the level of agreement between the SUCRA values (*bottom left*) obtained from the five methods used to synthesise evidence from network meta-analyses. Note that the data pictured have been simulated for illustrative purposes

**Fig. 4:**
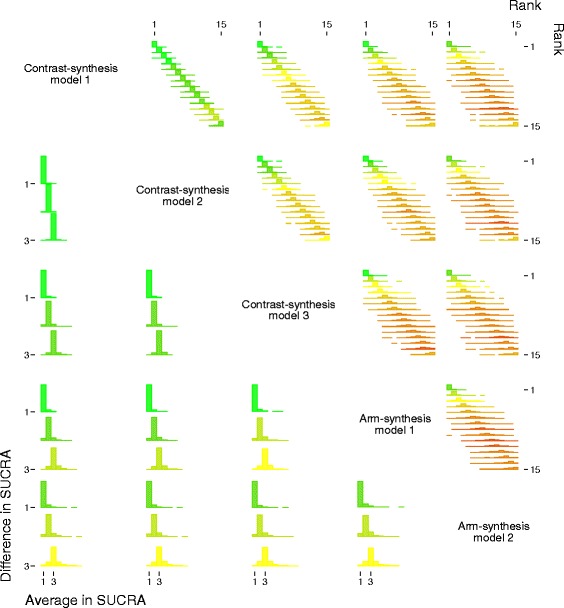
Comparison of rankings obtained from the five methods used to synthesise evidence from network meta-analyses. Note that the data pictured have been simulated for illustrative purposes

We will present graphical displays of the data that compare network estimates between the methods (described following). For illustrative purposes, Figs. 2, 3, and 4 depict results from simulated data, which were not derived from or related to the networks included in our empirical analysis.

We will graph the log of the odds ratios and 95% confidence/credible interval estimated from each method for each comparison within each of the networks. Figure 1 displays an example from a network that is not included in our cohort. Bland-Altman plots (Fig. 2) will be presented to assess the agreement between the estimates of the log of the odds ratios (upper corner of Fig. 2) and standard errors of the log of the odds ratios (lower corner of Fig. 2) using the five methods. If there is good agreement between methods, we would expect to see the differences between the methods scattered around the line of no difference (i.e., *y*-axis = 0), and most of the differences lying within two standard deviations of the mean difference [32, 33]. We will also use Bland-Altman plots to assess the agreement between SUCRA estimates (e.g. lower corner of Fig. 3).

To compare ranks, we will graphically display the agreement between the ranks obtained from each method as a proportion of the total number of treatments for each rank (Fig. 4 and upper corner of Fig. 3). For example, the first row, second column of the upper corner of Fig. 4, shows the comparison of ranks between the contrast-synthesis models 1 and 2. The bars in the diagonal show the proportion of times the first and second methods yielded the same result. In the off-diagonal, the proportion of times the first method ranked a treatment as *x* and the second method ranked that same treatment as *y* is displayed (where *x* = 1, 2, …, 20, and *y* = 1, 2, …, 20, but *x* $\ne$ *y*). In the lower corner of Fig. 4, we show the proportion of agreement between the methods for the first three rankings (i.e. *x* = 1, 2, and 3). These plots will provide a visual impression of the degree of agreement between the ranks estimated from each method, with greater spread and colour variation indicating less agreement. For example, in Fig. 4, first row, second column, the dominance of green bars with little variation indicates good agreement in the rankings between contrast-synthesis models 1 and 2, as compared with the first row, fourth column, where there is a lot of spread and colour variation, indicating a lack of agreement in the rankings between contrast-synthesis model 1 and arm-synthesis model 1.

The plots will be created overall and using different shades to identify the network characteristics defined above.

## Discussion

To our knowledge, this will be the first empirical study to compare a range of NMA methods applied to a large number of networks with binary outcomes. We aim to examine how the contrast-synthesis and arm-synthesis models compare; how the contrast-synthesis models in a frequentist and Bayesian framework compare; and how the results of the contrast-synthesis (Bayesian) models might be affected when assuming different prior distributions for the between-trial heterogeneity variance.

There are several strengths of our study. We are using a large number of networks to compare the methods. Therefore, we will be able to conclude with reasonable confidence whether in practice the choice of NMA method matters (for those methods evaluated). Further, following the lead of others [34, 35], we are publishing a protocol of our methodological study with the aim of clearly reporting and pre-specifying the objectives, design, and methods.

However, our study is not without limitations. We will focus on NMA with binary outcomes, so our findings may not be generalisable to other outcome types, such as continuous outcomes. While we are examining five models, many other models could be fitted, for example, a contrast-synthesis model with random study main effects or a contrast- or arm-synthesis model with unequal correlations between the random effects. Further, in our evaluation of the contrast-synthesis model in a Bayesian framework, we will use the between-trial heterogeneity variance priors from Turner et al. [16]. For a particular network, there may be a set of available priors (e.g. networks including pharmacological and non-pharmacological interventions) and we will select the prior that has the largest between-trial heterogeneity variance. We have chosen this approach because it is conservative, but other choices may impact on the findings. We are not aware of a set of prior values based on empirical evidence that can be used to vary the prior on the common heterogeneity variance in the arm-synthesis models, so we have limited our investigation of the arm-synthesis models to ones where the prior is fixed. Finally, we will limit the dataset to networks that demonstrate consistency. Although this consistency requirement will reduce the number of included networks, networks with identified inconsistency add to the complexity of the synthesis and are beyond the scope of this empirical study [4].

The use of NMA to synthesise direct and indirect evidence across a network of treatments is becoming increasingly popular because of the ability to address the important question of which treatment, from a set of treatments, is most effective. However, there is ongoing debate as to the most appropriate method to analyse these networks. This research will provide evidence on whether the choice of method in practice is important and may usefully inform the design of future simulation studies to investigate the performance of NMA methods.

## References

[1] Salanti G (2012). Indirect and mixed-treatment comparison, network, or multiple-treatments meta-analysis: many names, many benefits, many concerns for the next generation evidence synthesis tool. Res Synth Methods.

[2] Salanti G, Higgins JP, Ades AE, Ioannidis JP (2008). Evaluation of networks of randomized trials. Stat Methods Med Res.

[3] Lee A (2014). Review of mixed treatment comparisons in published systematic reviews shows marked increase since 2009. J Clin Epidemiol.

[4] Efthimiou O, Debray TP, Van Valkenhoef G, Trelle S, Panayidou K, Moons KG, Reitsma JB, Shang A, Salanti G, GetReal Methods Review G. GetReal in network meta-analysis: a review of the methodology. Res Synth Methods. 2016; 7(3): 236-263 doi:10.1002/jrsm.1195. 10.1002/jrsm.1195 26754852

[5] Mavridis D, Giannatsi M, Cipriani A, Salanti G (2015). A primer on network meta-analysis with emphasis on mental health. Evid Based Ment Health.

[6] Salanti G, Marinho V, Higgins JP (2009). A case study of multiple-treatments meta-analysis demonstrates that covariates should be considered. J Clin Epidemiol.

[7] Neupane B, Richer D, Bonner AJ, Kibret T, Beyene J (2014). Network meta-analysis using R: a review of currently available automated packages. PLoS One.

[8] Dias S, Ades AE (2016). Absolute or relative effects? Arm-based synthesis of trial data. Res Synth Methods.

[9] Hong H, Carlin BP, Shamliyan TA, Wyman JF, Ramakrishnan R, Sainfort F, Kane RL (2013). Comparing Bayesian and frequentist approaches for multiple outcome mixed treatment comparisons. Med Decis Making.

[10] Hong H, Chu H, Zhang J, Carlin BP (2016). A Bayesian missing data framework for generalized multiple outcome mixed treatment comparisons. Res Synth Methods.

[11] Thorlund K, Thabane L, Mills EJ (2013). Modelling heterogeneity variances in multiple treatment comparison meta-analysis—are informative priors the better solution?. BMC Med Res Methodol.

[12] Hong H, Chu H, Zhang J, Carlin BP (2016). Rejoinder to the discussion of “a Bayesian missing data framework for generalized multiple outcome mixed treatment comparisons,” by S. Dias and A. E. Ades. Res Synth Methods.

[13] Zhang J, Carlin BP, Neaton JD, Soon GG, Nie L, Kane R, Virnig BA, Chu H (2014). Network meta-analysis of randomized clinical trials: reporting the proper summaries. Clin Trials.

[14] Lu G, Ades AE (2004). Combination of direct and indirect evidence in mixed treatment comparisons. Stat Med.

[15] Higgins JP, Whitehead A (1996). Borrowing strength from external trials in a meta-analysis. Stat Med.

[16] Turner RM, Davey J, Clarke MJ, Thompson SG, Higgins JP (2012). Predicting the extent of heterogeneity in meta-analysis, using empirical data from the Cochrane Database of Systematic Reviews. Int J Epidemiol.

[17] Turner RM, Jackson D, Wei Y, Thompson SG, Higgins JP (2015). Predictive distributions for between-study heterogeneity and simple methods for their application in Bayesian meta-analysis. Stat Med.

[18] Burton A, Altman DG, Royston P, Holder RL (2006). The design of simulation studies in medical statistics. Stat Med.

[19] Song F, Clark A, Bachmann MO, Maas J (2012). Simulation evaluation of statistical properties of methods for indirect and mixed treatment comparisons. BMC Med Res Methodol.

[20] Langan D, Higgins JP, Simmonds M (2015). An empirical comparison of heterogeneity variance estimators in 12 894 meta-analyses. Res Synth Methods.

[21] Petropoulou M, Nikolakopoulou A, Veroniki AA, Rios P, Vafaei A, Zarin W, Giannatsi M, Sullivan S, Tricco AC, Chaimani A, Egger M, Salanti G (2017). Bibliographic study showed improving statistical methodology of network meta-analyses published between 1999 and 2015. J Clin Epidemiol.

[22] White IR (2015). Network meta-analysis. Stata Journal.

[23] Veroniki AA, Mavridis D, Higgins JP, Salanti G (2014). Characteristics of a loop of evidence that affect detection and estimation of inconsistency: a simulation study. BMC Med Res Methodol.

[24] Zhang J, Chu H, Hong H, Virnig BA, Carlin BP. Bayesian hierarchical models for network meta-analysis incorporating nonignorable missingness. Stat Methods Med Res. 2015. doi:10.1177/0962280215596185. 10.1177/0962280215596185 PMC4731325 26220535

[25] Lambert PC, Sutton AJ, Burton PR, Abrams KR, Jones DR (2005). How vague is vague? A simulation study of the impact of the use of vague prior distributions in MCMC using WinBUGS. Stat Med.

[26] Rucker G (2012). Network meta-analysis, electrical networks and graph theory. Res Synth Methods.

[27] Schwarzer G, Carpenter JR, Rücker G. Meta-Analysis with R: Springer International Publishing; 2015.

[28] Trinquart L, Attiche N, Bafeta A, Porcher R, Ravaud P (2016). Uncertainty in treatment rankings: reanalysis of network meta-analyses of randomized trials. Ann Intern Med.

[29] Brooks S, Gelman A (1998). Some issues in monitoring convergence of iterative simulations. Journal of Computational and Graphical Statistics.

[30] Brooks S, Gelman A (1998). General methods for monitoring convergence of iterative simulations. Journal of Computational and Graphical Statistics.

[31] Salanti G, Ades AE, Ioannidis JP (2011). Graphical methods and numerical summaries for presenting results from multiple-treatment meta-analysis: an overview and tutorial. J Clin Epidemiol.

[32] Bland JM, Altman DG (1986). Statistical methods for assessing agreement between two methods of clinical measurement. Lancet.

[33] Bland JM, Altman DG (1999). Measuring agreement in method comparison studies. Stat Methods Med Res.

[34] Akl EA, Briel M, You JJ, Lamontagne F, Gangji A, Cukierman-Yaffe T, Alshurafa M, Sun X, Nerenberg KA, Johnston BC, Vera C, Mills EJ, Bassler D, Salazar A, Bhatnagar N, Busse JW, Khalid Z, Walter S, Cook DJ, Schunemann HJ, Altman DG, Guyatt GH (2009). LOST to follow-up Information in Trials (LOST-IT): a protocol on the potential impact. Trials.

[35] Sun X, Briel M, Busse JW, Akl EA, You JJ, Mejza F, Bala M, Diaz-Granados N, Bassler D, Mertz D, Srinathan SK, Vandvik PO, Malaga G, Alshurafa M, Dahm P, Alonso-Coello P, Heels-Ansdell DM, Bhatnagar N, Johnston BC, Wang L, Walter SD, Altman DG, Guyatt GH (2009). Subgroup Analysis of Trials Is Rarely Easy (SATIRE): a study protocol for a systematic review to characterize the analysis, reporting, and claim of subgroup effects in randomized trials. Trials.

